# Brain Activity and Connectivity in Fatigued People with Multiple Sclerosis During a Static Balance Task: An fNIRS Study

**DOI:** 10.3390/s26165131

**Published:** 2026-08-13

**Authors:** Davide Cattaneo, Alessandro Torchio, Augusto Bonilauri, Chiara Corrini, Nora E. Fritz, Francesca Baglio, Elisa Gervasoni

**Affiliations:** 1 IRCCS Fondazione Don Carlo Gnocchi, 20148 Milan, Italy; davide.cattaneo@unimi.it (D.C.); atorchio@dongnocchi.it (A.T.); ccorrini@dongnocchi.it (C.C.); egervasoni@dongnocchi.it (E.G.); 2 Department of Pathophysiology and Transplantation, University of Milan, 20100 Milan, Italy; 3 Department of Electronics, Information and Bioengineering, Politecnico di Milano, 20133 Milan, Italy; augusto.bonilauri@polimi.it; 4 Department of Health Care Sciences and Neurology, Wayne State University, Detroit, MI 48201, USA; nora.fritz@wayne.edu

**Keywords:** functional near-infrared spectroscopy, fatigue, multiple sclerosis, balance

## Abstract

Fatigue and balance disorders are common in people with multiple sclerosis (PwMS), but the neural mechanisms linking them during postural control remain unclear. This cross-sectional study aims to examine associations between fatigue, balance impairment, and task-evoked cortical hemodynamics/connectivity. Sixteen PwMS (relapsing-remitting MS; EDSS < 3.5) and 19 healthy controls (HC) were enrolled. Based on the Modified Fatigue Impact Scale (MFIS), PwMS were classified as fatigued (f_PwMS, MFIS ≥ 38; *n* = 10) or non-fatigued (nf_PwMS, *n* = 6). Participants performed five 30 s trials of static stance on foam with eyes closed. fNIRS recorded frontal, temporal, and occipital cortical activity, while stabilometry measured center-of-pressure sway. In PwMS, MFIS showed a trend toward association with sway (r = 0.49; *p* = 0.06). Sway was higher in f_PwMS than nf_PwMS and HC but not significantly different (ANOVA *p* = 0.341). Compared with HC, PwMS showed increased bilateral prefrontal and reduced occipital HR. Stratified analyses showed greater HR in f_PwMS versus HC and nf_PwMS in bilateral BA10 and in temporo-parietal regions, with a right-hemisphere predominance. Global coherence did not differ across groups, but exploratory BA10 analysis showed higher prefrontal coherence in f_PwMS than nf_PwMS (*p* = 0.02). Fatigued PwMS exhibit amplified, right prefrontal and temporo-parietal activation and focal prefrontal hyper-connectivity, consistent with compensatory top–down control to maintain balance.

## 1. Introduction

Fatigue, defined as a subjective lack of physical and/or mental energy [1], is one of the most disabling symptoms of multiple sclerosis (MS) [2], affecting 50–90% of people with MS (PwMS) [3]. Neuroimaging studies link fatigue to lower gray-matter volumes in frontal, temporal, and parietal cortices and to alterations within cortico-striato-thalamo-cortical circuits [4]. Frontal cortical changes have been associated with subjective fatigue and cognitive fatigability. Trait fatigue also correlates with lesion load in frontal–parietal pathways and atrophy in bilateral middle and superior frontal gyri [4]. Together, these findings suggest that fatigue reflects increased neural effort to maintain performance in the MS-affected brain, especially during complex tasks [5,6].

Balance disorders are also common in PwMS. Stabilometric studies show that about three-quarters of PwMS have abnormal postural control with eyes open, and almost all do when visual and proprioceptive inputs are reduced, such as during eyes-closed stance on foam [7]. Moreover, hypointense T1 lesions and lesion burden in superior/medial frontal and temporal lobes and the parietal operculum have been associated with impaired stance during sensory manipulation [8]. Larger postural sway with eyes closed (EC) has also been related to the damage of specific structures and to disruptions in networks subserving balance control [9].

Structural and functional brain changes in MS have been associated with balance deficits, fatigue and fatigability [10,11,12]. Functional involvement of cerebellar and brainstem systems has been linked to fatigue in a study measuring static balance under various sensory conditions [13]. However, fatigue’s effect on balance remains unclear: some studies report links between trait fatigue and standing balance across sensory conditions, while others find no balance changes after fatiguing tasks [11,14]. Existing studies have generally assessed balance, fatigue, and brain dysfunction separately. Concurrent assessment of neural dynamics and postural control may clarify the central mechanisms of balance in PwMS. Functional near-infrared spectroscopy (fNIRS) is a non-invasive method to quantify cortical hemodynamics during standing tasks [15]. Combined with stabilometric platforms, it enables simultaneous measurement of cortical activity, postural sway, and their associations with motor fatigue [16,17].

Thus, the aim of this study was to investigate the relationship between cortical activity, balance impairments, and fatigue in PwMS. We hypothesized that cortical activity would be greater in fatigued PwMS during a balance task requiring sensory reweighting than non-fatigued PwMS. Further, we hypothesized that increased cortical activity in fatigued PwMS would be coupled with reduced connectivity.

## 2. Materials and Methods

### 2.1. Study Design

This cross-sectional study was approved by the Ethics Committee of IRCCS Fondazione Don Carlo Gnocchi, Milan, Italy (ID protocol code: 20190119; date of approval: 19 October 2020) and conducted in accordance with the Declaration of Helsinki. All participants provided written informed consent prior to enrolment. This manuscript was prepared in accordance with the Strengthening the Reporting of Observational Studies in Epidemiology (STROBE) statement.

### 2.2. Participants

We recruited 16 right-handed people with MS (PwMS) and 19 healthy controls (HC) between January 2021 and January 2023. Inclusion criteria for PwMS included relapsing–remitting MS; age > 17 years; no relapses or pharmacological changes within 3 months; Expanded Disability Status Scale (EDSS) < 3.5; and ability to stand independently on foam with EC for 30 s. Exclusion criteria included inability to understand/sign informed consent; peripheral vestibular disorders; diagnosed psychiatric or cognitive disorders; musculoskeletal/orthopedic conditions; other neurological disorders; or severe visual impairment.

### 2.3. Clinical Assessment

Before the experimental protocol, demographic and clinical data were collected, and participants completed the following assessment: mini-Balance Evaluation Systems Test (mini-BESTest) [18] to assess balance performance; Activity-specific Balance Confidence Scale (ABC) to assess confidence in performing activities without losing balance [19]; and the Timed 25-Foot Walk (T25FW) to measure walking speed [20]. Trait fatigue was the primary outcome and was assessed with the 21-item Modified Fatigue Impact Scale (MFIS). Items are rated 0 (“Never”) to 4 (“Almost always”), with higher scores indicating greater fatigue. The established cut-off of 38 points classified participants as fatigued or non-fatigued [21].

### 2.4. Stabilometric Assessment

Postural control was measured with a stabilometric platform (Prokin 252, TecnoBody^©^, Dalmine, Italy) providing instantaneous center-of-pressure (CoP) positions at 20 Hz. Tests were performed in a quiet room with dim lighting to minimize interference with fNIRS. Participants wore their usual shoes and clothing. After familiarization, the experimental condition was standing, as still as possible, with EC on foam for five times (each 30 s). A baseline condition, eyes open (EO) on foam, while fixating a white cross on a black screen centered at eye level, was performed after the EC condition to allow hemodynamic response (HR) to return to the baseline level. The primary sway metric (postural sway) was the center-of-pressure (CoP) path length (Perimeter, mm), computed from the stabilometric data.

### 2.5. fNIRS Acquisition

**Figure 1:**
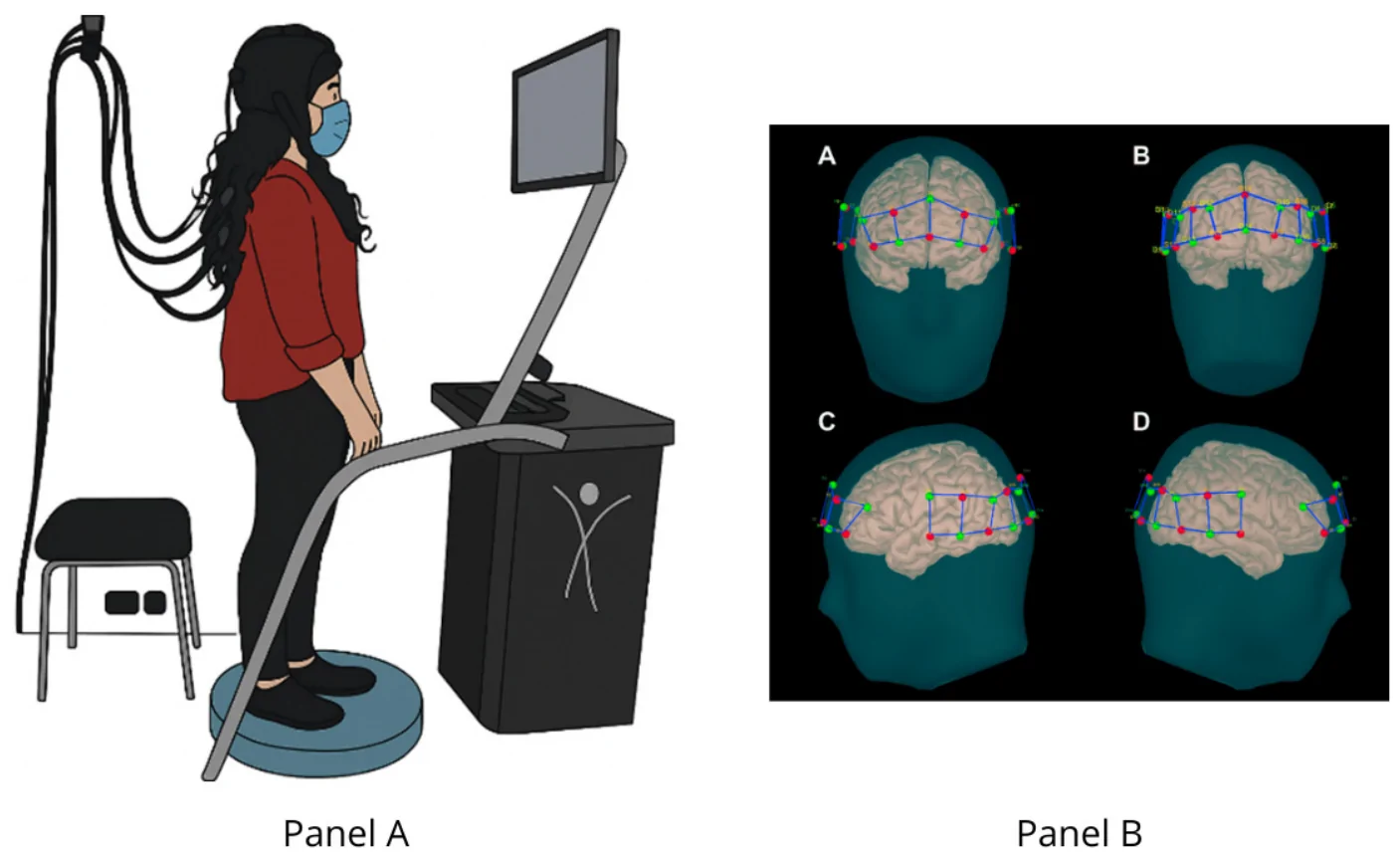
Experimental setup and channel locations. (**Panel A**): Experimental setup. (**Panel B**): Schematic locations of fNIRS sources (red), detectors (green), and measurement channels connecting source–detector pairs (blue lines) over cortical anatomy: A: frontal cortex; B: occipital cortex; C: temporal cortex (left hemisphere); D: temporal cortex (right hemisphere).

Cortical hemodynamics during the eyes-closed-on-foam balance task were recorded with a continuous-wave fNIRS system (NIRScoutX, NIRx Medizintechnik, Berlin, Germany) at 760 and 850 nm. Signals were sampled at 3.91 Hz using 16 LED sources and 16 avalanche photodiode detectors. A qualified fNIRS researcher positioned optodes according to the international 10/5 system. The probe comprised 44 measurement channels with a mean ± SD source–detector distance of 2.73 ± 0.72 cm. The montage targeted major frontal, temporal, and occipital cortices (Figure 1). A whole-head setup was avoided to reduce burden during upright testing. The selection emphasized temporo-parietal regions implicated in balance control under vestibular conditions (foam-EC) and frontal regions for their modulatory role [15,22]. Moreover, the inclusion of occipital areas enabled exploration of sensory integration processes when visual input is altered. Supplementary Material S1 reports MNI coordinates for each channel center and corresponding Brodmann areas (BAs). Following the setup, participants performed the stabilometric protocol as described above.

### 2.6. fNIRS Preprocessing and Analysis

The fNIRS data were preprocessed for obtaining averaged responses to foam-EC conditions. Data preprocessing was performed through Homer2 [23], and BRAIN AnalyzIR toolboxes in Matlab R2022b (The MathWorks, Inc., Natick, MA, USA) [24]. Details of the employed data processing pipeline are described in a previous study [25]. Briefly, measurement channels with a coefficient of variation lower than 10% were firstly excluded from further analyses, due to an insufficient signal-to-noise ratio (SNR) level. Next, measurements were respectively converted into optical density data, corrected for motion artifact through a wavelet-based method, bandpass-filtered in [0.01–0.3] Hz and corrected for systemic physiological interference by removing the first PCA component across channels. Hence, variations in concentrations were obtained according to modified Beer–Lambert law and averaged across the 30 s time window to obtain an estimate of hemodynamic response (HR).

### 2.7. Data Analysis

Correlation between fatigue and balance (Perimeter) was assessed by Spearman’s correlation coefficient. Subsequently, PwMS were categorized as non-fatigued (nf_PwMS) if MFIS <38 points and fatigued (f_PwMS) if MFIS ≥ 38 points. To characterize balance changes, we compared Perimeter across HC, nf_PwMS, and f_PwMS using one-way ANOVA. A linear mixed-effects model was fitted with the time series of HR (μM) as the dependent variable. Fixed effects were Brodmann area (e.g., BA10, BA21–22, and BA40), Group (HC, nf_PwMS, and f_PwMS), and Side (left/right hemisphere). A random intercept for Subject captured between-participant variability, and a first-order autoregressive [AR(1)] covariance structure modeled temporal dependence of the time series. Model performance was summarized by Nagelkerke’s pseudo-R^2^ and the root-mean-square error (RMSE, μM) computed from fixed-effect residuals. Post hoc contrasts used estimated marginal means with Tukey adjustment. We first contrasted HC vs. PwMS to align with the prior literature, then performed pairwise comparisons among HC, nf_PwMS, and f_PwMS. As additional analysis, we also contrasted HC with the two subgroups (f_PwMS and nf_PwMS) together.

To investigate associations among fatigue, balance, and HR, we examined the bivariate association between MFIS and Perimeter. Next, we added to the preceding mixed model the Perimeter as predictor, calculating between-group differences and differences in slopes of the association between HR and Perimeter. Sex and age were initially entered as covariates but then removed from subsequent models since they were not significantly associated with HR.

Finally, to examine the functional connectivity, inter-channel connectivity was estimated using coherence analysis to quantify stable amplitude/phase relationships among channels within each group using the coh function in the seewave package in R version 4.6. Group comparisons were performed using a mixed model with between-channel pair coherence as a dependent variable, group as a fixed effect with three levels (HC, f_PwMS and nf_PwMS) and subject as a random variable.

## 3. Results

### 3.1. Sample

Sixteen PwMS were included in the analyses (female *n* = 15, 90%). All had relapsing–remitting MS and were mostly mildly disabled (EDSS, median [IQR]: 2.00 [1.00]). Mean (±SD) age was 43.2 ± 11.0 years; disease duration was 8.9 ± 7.2 years. Nineteen healthy controls (HC; female *n* = 13, 68.4%), with a mean age of 37.0 ± 9.0 years, were included. The association between MFIS and postural sway (Perimeter) in the whole MS group approached significance (r = 0.49; *p* = 0.06).

### 3.2. Fatigue Subgroups

Clinical and demographic characteristics for fatigued (f_PwMS, *n* = 10) and non-fatigued (nf_PwMS, *n* = 6) participants are reported in Table 1. MFIS scores were 17.3 ± 10.0 (nf_PwMS) and 48.2 ± 8.4 points (f_PwMS). The f_PwMS group was older and more disabled compared to nf_PwMS. Postural sway was higher in f_PwMS (mean ± SD: 1305.0 ± 175.6 mm) than in nf_PwMS (1223.1 ± 124.2 mm) and HC (1055.2 ± 90.1 mm), but between-group differences were not significant (ANOVA: F = 1.114; *p* = 0.341).

### 3.3. Differences Between HC and PwMS

**Figure 2:**
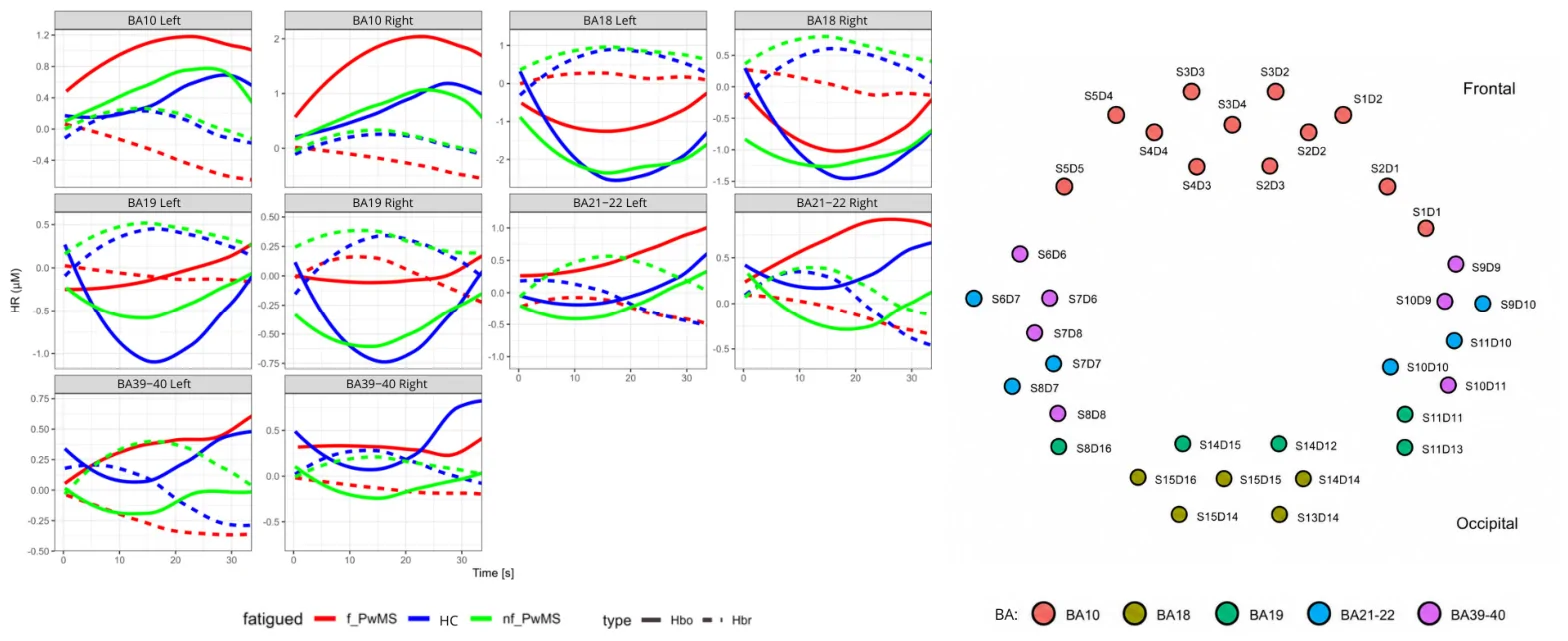
Time series of oxy- and deoxygenated hemoglobin and representation of the channel set. HR: hemodynamic response; BA: Brodmann area; f_PwMS: fatigued people with multiple sclerosis; nf_PwMS: non-fatigued people with multiple sclerosis; HC: healthy controls; HbO: oxy-hemoglobin; HbR: deoxy-hemoglobin. Noisy channels, removed from statistical analysis, are omitted in the right plot. Noisy channels with high SNR were excluded from analysis and they are omitted in the graphical representation.

Figure 2 shows time series of oxygenated/deoxygenated hemoglobin by BA. Visual inspection suggested condition-related HR changes in HC and in both MS subgroups over the 30 s trial.

The mixed model showed statistically significant main effects and interactions (Supplementary Material S2), with excellent fit (conditional-R^2^ = 0.91) and RMSE = 1.5 µM.

In the first preliminary comparison, larger HR changes were found (PwMS–HC, estimated difference: est.diff) in PwMS compared with HC bilaterally in frontal areas BA10 (right, est.diff = 0.28 ± 0.08 µM; t = 3.1; *p* = 0.002; left, est.diff = 0.25 ± 0.08 µM; t = 3.0; *p* = 0.004). Further, there was also reduced HR bilaterally in occipital areas BA18 (right, est.diff= −0.20 ± 0.08 µM; t = −2.4; *p* = 0.02; left, est.diff= −0.34 ± 0.09 µM; t = −4.0; *p* = 0.0003) in PwMS compared to HC.

### 3.4. Differences Between HC, nf_PwMS, and f_PwMS

**Figure 3:**
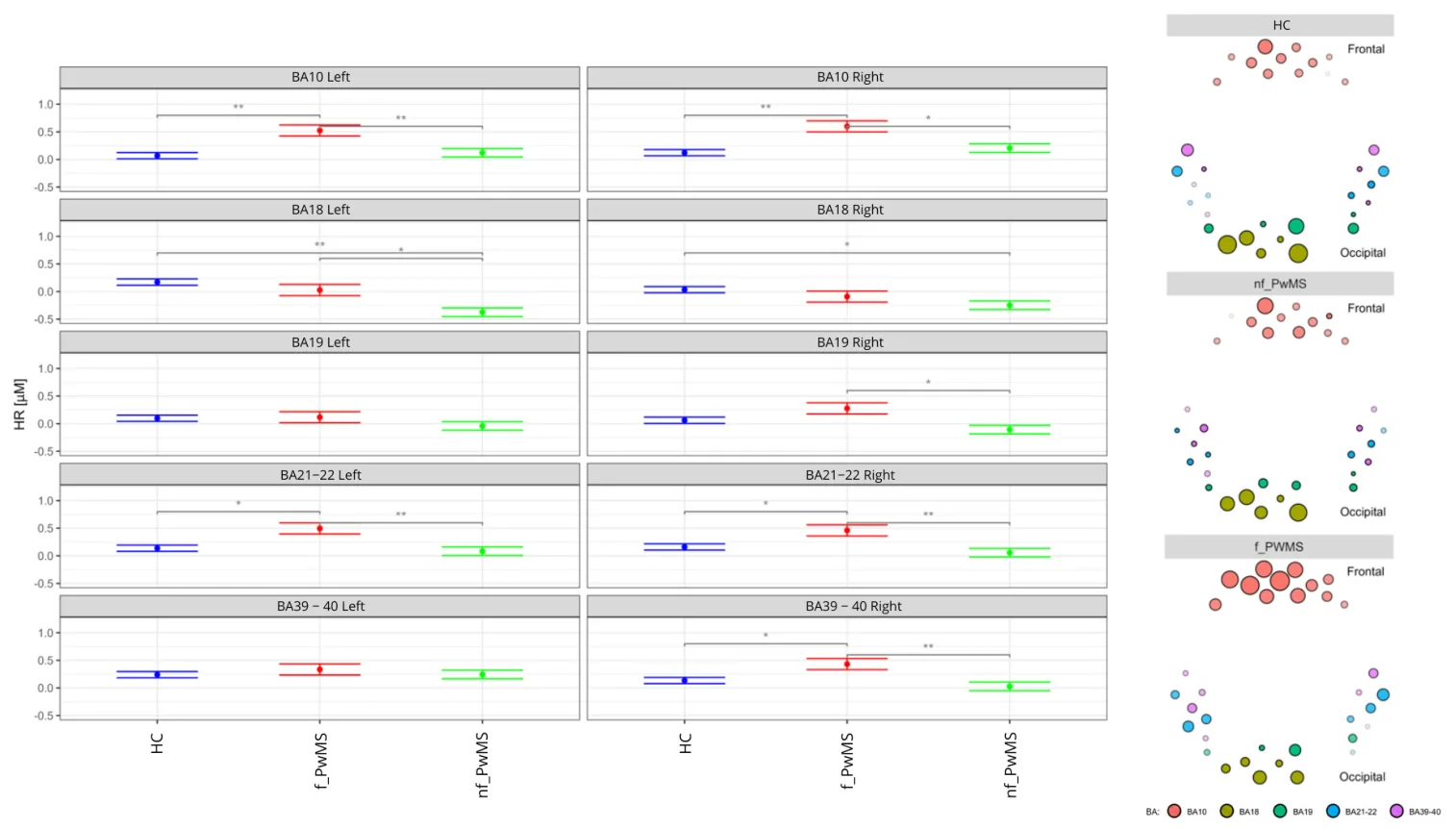
HR in the three groups and the topographical representation of channel positions and their corresponding BA. HC, healthy controls; nf_PwMS, non-fatigued people with multiple sclerosis; f_PwMS, fatigued people with multiple sclerosis. The circle diameter reflects the magnitude of HR. Colored diamonds indicate the estimated marginal mean HR for each group (blue = HC, red = f_PwMS, green = nf_PwMS), and the horizontal bars represent the 95% confidence intervals. * *p* < 0.05. ** *p* < 0.001. Tukey method for multiple comparisons.

Figure 3 depicts estimated means of HR in HC, f_PwMS, and nf_PwMS, together with the topographical arrangement of channels and their corresponding BA. Comparing HC and f_PwMS, significant differences in left and right BA10 (HC–f_PwMS, right, est.diff= −0.47 ± 0.12 µM; t = −4.2; *p* < 0.001; left: est.diff = −0.45 ± 0.11 µM; t = −4.0; *p* < 0.001) were observed. Specifically, HR was nearly eight-fold larger in f_PwMS than HC in right BA10, while the ratio was ~5 in left BA10. Additional bilateral differences emerged in BA21–22 (HC–f_PwMS, right, est.diff= −0.29 ± 0.11 µM; t = −2.6; *p* = 0.04; left: est.diff = −0.35 ± 0.16 µM; t = −3.1; *p* = 0.011). Overall, right-hemisphere activity appeared greater, with further activation in right BA 39–40 (HC–f_PwMS, right, est.diff = −0.30 ± 0.12 µM; t = −2.6; *p* = 0.04). The comparison between f_PwMS and nf_PwMS showed a similar pattern, with the exception that left BA 18 and right BA 19 also differed between groups. Notably, an unexpected pattern was observed in BA 18 bilaterally and in right BA 19, with smaller concentrations in nf_PwMS compared with HC.

### 3.5. Differences Between HC, nf_PwMS, and f_PwMS and Associations with Balance Disorders

**Figure 4:**
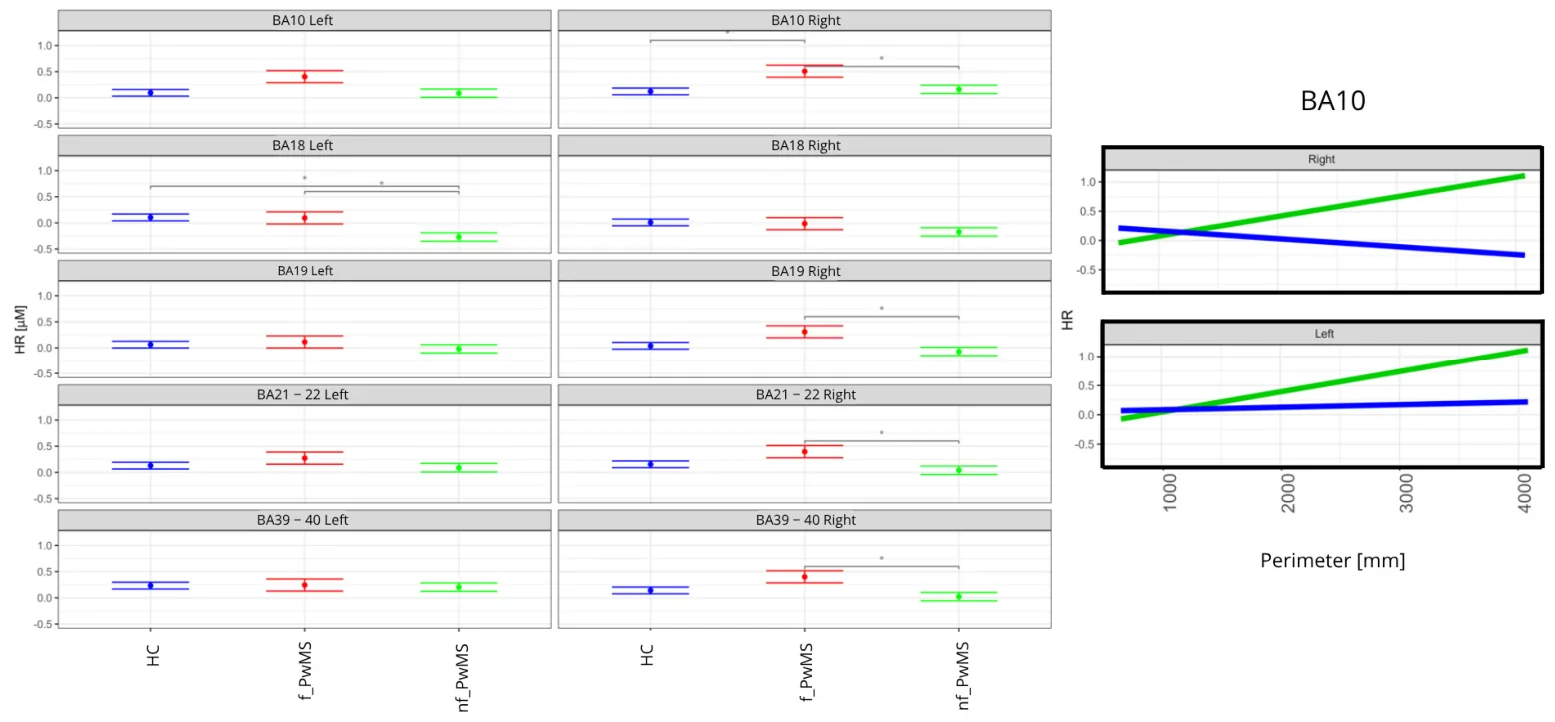
HR in the three subgroups and the relationship between Perimeter and HR by hemisphere and Brodmann area. Colored diamonds indicate the estimated marginal mean HR for each group (blue = HC, red = f_PwMS, green = nf_PwMS), and the horizontal bars represent the 95% confidence intervals. HC = healthy subjects; nf_PwMS = non-fatigued people with multiple sclerosis; f_PwMS = fatigued people with multiple sclerosis. In the right panel, blue = HC + nf_PwMS, green = f_PwMS. * *p* < 0.05.

We examined whether group differences in HR persisted after accounting for balance and how HR related to postural sway (Figure 4). Model fit was comparable to the preceding analyses (Supplementary Material S3) (conditional-R^2^ = 0.91; RMSE = 1.5 µM). After adjusting for body postural, f_PwMS showed greater HR than HC in right BA10, with the contrast HC−f_PwMS equal to −0.38 ± 0.13 µM; t = 2.9; *p* = 0.01 (Figure 4, left).

Comparing f_PwMS vs nf_PwMS, we found higher HR in f_PwMS for left BA18 (est.diff = 0.36 ± 0.14 µM; t = 2.6; *p* = 0.04), right BA10 (est.diff = 0.34 ± 0.14 µM; t = 2.47; *p* = 0.04), right BA19 (est.diff = 0.37 ± 0.14 µM; t = 2.7; *p* = 0.02), right BA21–22 (est.diff = 0.35 ± 0.14 µM; t = 2.5; *p* = 0.040), and right BA39–40 (est.diff = 0.37 ± 0.14 µM; t = 2.7; *p* = 0.022).

Moreover, HC group presented a significant higher activation in left BA18 compared to nf_PwMS (HC−nf_PwMS, est. diff = 0.37 ± 0.10 µM; t = 3.6; *p* = 0.003). The model yielded a significant BA × side × group × Perimeter interaction (*p* < 0.0001), indicating that worsening balance control was associated with HR changes that depended on brain area, hemisphere, and group. The model also showed between-group slope difference (Figure 4, right plots). No between-group differences in slope survived adjusting for multiple comparisons. An explorative post hoc contrast combining HC and nf_PwMS vs. f_PwMS showed a slope difference in right BA10 (pairwise *p* = 0.04), but not in left BA10 (*p* = 0.19).

**Figure 5:**
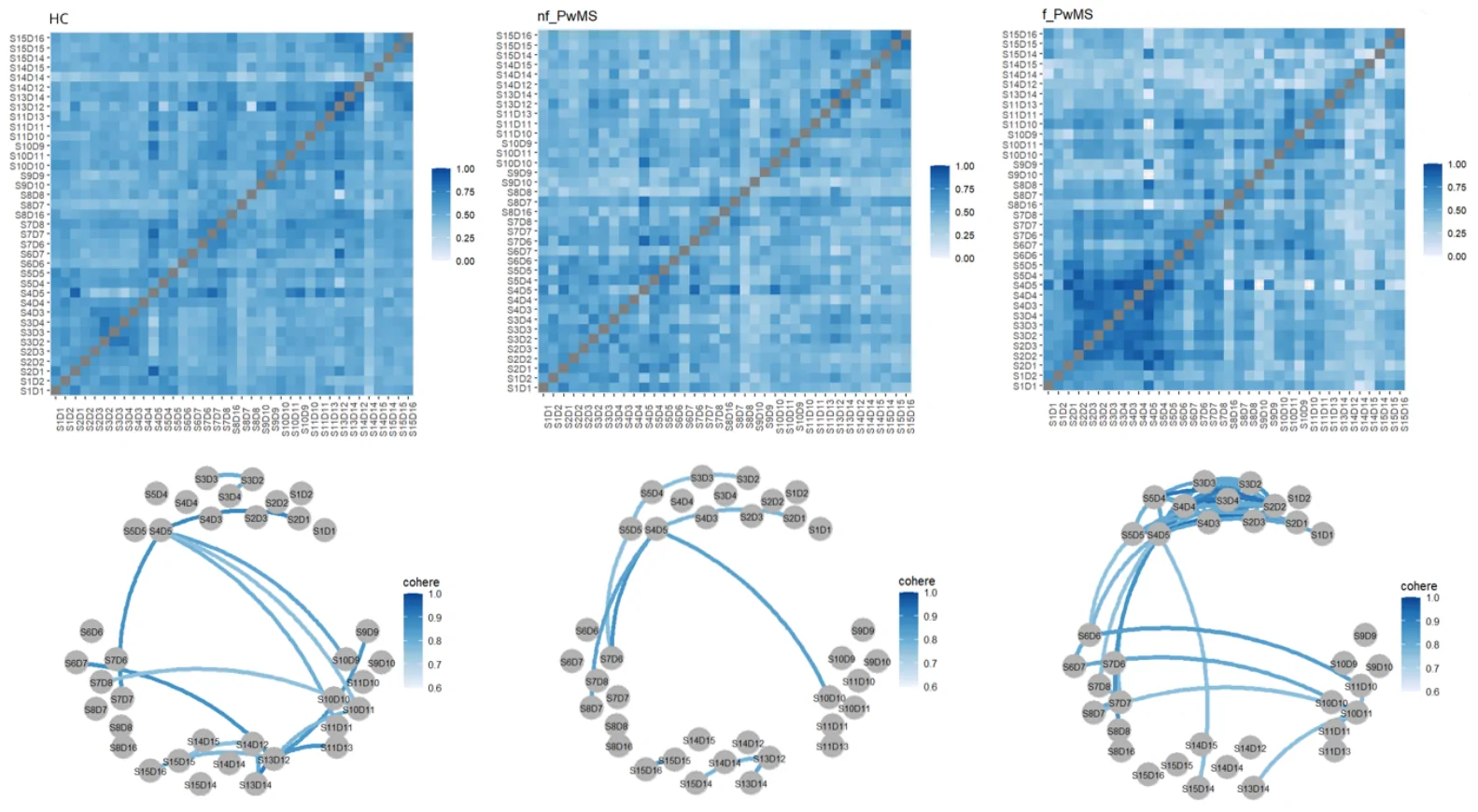
Channel-by-channel coherence analysis for HC, nf_PwMS, f_PwMS and topological representation. HC: healthy subjects; nf_PwMS: non-fatigued people with multiple sclerosis; f_PwMS: fatigued people with multiple sclerosis. Note that we represent only coherence values above 0.75 to improve clarity. Darker edge colors represent larger values of coherence. Gray cells along the diagonal of each coherence matrix represent self-coherence values (i.e., the coherence of each channel with itself) and are therefore not considered in the analysis.

Connectivity across channels is shown in Figure 5. When all channels were considered, HC exhibited higher mean coherence than both MS groups (HC: 0.54 ± 0.02 au; nf_PwMS: 0.50 ± 0.02 au; f_PwMS: 0.50 ± 0.03 au), However, no statistically significant between-group differences were present (F = 2.02; *p* = 0.15). Visual inspection revealed bilateral frontal connectivity approximating left and right BA10. An exploratory analysis of both BA10 showed a significant group effect (F = 3.40; *p* = 0.03): f_PwMS displayed higher coherence (0.85 ± 0.07 au) than HC (0.68 ± 0.04 au) and nf_PwMS (0.58 ± 0.06 au), with a significant difference between f_PwMS and nf_PwMS (Δ = 0.26 ± 0.09; t = 2.81; *p* = 0.02).

## 4. Discussion

PwMS showed higher cortical activity than HC during a balance task that challenges sensory conditions, specifically in the frontal and temporal regions, which are involved in the sensorimotor circuit. This finding seems to corroborate the hypothesis that HR increases during challenging tasks in PwMS, which has also been found in divided-attention locomotion [26] and dual-task paradigms [27]. Likewise, increased brain activity in complex sensory conditions has been reported in Parkinson’s disease [28], stroke [29], and older adults, specifically in the parietal and prefrontal regions [30,31].

In our study, the difference in cortical activation became more pronounced when the MS group was divided into f_PwMS and nf_PwMS. We found enhanced bilateral prefrontal cortex activation in f_PwMS during the balance task. This may reflect a compensatory mechanism involving higher-order brain systems to integrate somatosensory, visual, and vestibular information, as well as supporting anticipatory movement. It may also indicate that balance control under this complex condition becomes more attention-demanding, requiring greater frontal engagement [6]. Higher activation in prefrontal cortex during balance tasks has been documented in persons with neurological disorders. Stroke survivors showed increased bilateral prefrontal activity in postural perturbation tasks along with the premotor and parietal regions [28]. Likewise, people with Parkinson’s disease demonstrated higher prefrontal activity to maintain postural stability [27]. Higher prefrontal activation may help maintain stability during demanding balance tasks. This frontal response does not seem task-specific, as it has also been reported during other motor and cognitive tasks in PwMS [32,33]. We evaluated trait fatigue, but changes in brain activity have also been documented with EEG following a cognitively fatiguing task in PwMS [34]. Increased fatigability was associated with increased fronto-medial theta and occipital alpha power. Researchers also found a shift from fast to slow-frequency waves, suggesting that compensatory mechanisms are in place to improve cognitive control processes following fatiguing tasks. Moreover, imaging studies demonstrate that functional changes observed in frontal regions are associated with lesion load, gray matter atrophy, and structural changes in cortical–subcortical pathways as in the cortico-striato-thalamo-cortical network [35], which cannot be assessed thoroughly by fNIRS.

At the network level, consistent with a previous study, we observed a trend toward lower mean global connectivity in both PwMS groups compared with HC [17]. However, contrary to our hypothesis, our exploratory analysis of BA10 revealed a focal increased connectivity in f_PwMS compared to the other two groups. While causal inference is limited and the results are from exploratory analysis, one interpretation is that prefrontal hubs transiently up-regulate local coupling to stabilize performance when sensorimotor networks are inefficient. In this view, higher subjective fatigue could reflect the resource cost of sustaining such top–down control in challenging balance contexts.

Our results also point toward greater temporo-parietal contributions and right-hemisphere predominance in f_PwMS with a greater activation in BA21–22, with a pronounced right-lateralized pattern. Prior studies in HC implicate temporo-parietal regions—superior temporal gyrus (STG) and supramarginal gyrus [15]—in processing distorted visual/proprioceptive cues and resolving sensory conflict; the supramarginal gyrus, in particular, is linked to proprioceptive integration [18]. Consistently, right frontal/parietal opercular and STG activations have been reported under similar conditions [22], again pointing to a right-hemisphere bias similar to our findings. Crucially, in our data these effects persisted after accounting for postural sway, suggesting that they are not explained solely by peripheral sensorimotor damage commonly observed in PwMS.

Balance is commonly impacted in PwMS, with impairments of the somatosensory, visual, and vestibular systems leading to altered sensory integration that is an essential component of balance control [7]. The relationship between HR and postural sway (Perimeter) was unclear. Although we detected an overall interaction (BA × side × group × Perimeter), only ad hoc effects survived multiple-comparison control, showing changes in HR in f_PwMS with larger balance disorders in frontal regions. This increment is in keeping with other studies reporting increased activity coupled with the recruitment of additional regions in HC when they are faced with more challenging balance tasks [29,30]. With this perspective, HR from the right side of the brain is a candidate biomarker to assess the effectiveness of balance interventions. A larger study adopting a multivariate framework combining postural sway metrics with regional HR could help to elucidate whether improvements in balance rehabilitation are due to compensatory or restorative mechanisms [36]. Compensatory mechanisms are reflected in improved postural control achieved through increased, atypical brain activity. In contrast, restorative mechanisms are associated with better postural control through a return toward more physiological brain activity, such as reduced cortical activation in parieto-temporal and frontal, together with a decreased connectivity in bilateral BA10.

Our working hypothesis is that higher prefrontal activation and increased local connectivity help maintain stance when lesions compromise the sensorimotor loop. This increased activation and connectivity of additional non-motor regions may contribute to increased perceived fatigue in MS. However, difficulties in data interpretation were evident when balance disorders were added as covariate. Thus, although correcting for observed confounders improves prediction, results remain speculative when it comes to inferring causality between fatigue and BA21–22 activity. It would be interesting to verify if, after treatment, a decrease in subjective fatigue is associated with decreased brain activity. To strengthen our results, it would also be important to recruit a larger sample to allow for accurate estimation of brain activity with confounders added to the model.

### Limitations

A primary limitation of this study is the small sample size, which reduces power and precision. Specifically, the model included a large number of fNIRS time points, while the number of participants was relatively limited, increasing the risk of false-positive findings and/or model overfitting. Therefore, the results should be interpreted primarily as model-estimated temporal patterns rather than evidence of statistically significant effects at individual time points. Secondly, the unequal sex distribution between the PwMS and HC groups may have introduced residual confounding. Although the analyses were adjusted for sex, the relatively small sample size limited our ability to fully investigate potential sex-related effects. Thirdly, cognitive impairment was not assessed, preventing analysis of cognition–balance–prefrontal relationship; cognitive fatigue may therefore have confounded some effects. Fourthly, spatial localization is constrained by fNIRS’ resolution and atlas-based approach, and the upright setup precluded coverage of primary sensorimotor areas most active during stance. Finally, the absence of short-separation channels limited our ability to fully separate cortical from superficial signals, although we implemented the principal component analysis (PCA), which is a well-established alternative preprocessing strategy when short-separation channels are unavailable [37,38]. Future studies should include both static and dynamic balance tasks, incorporate short channels and broader sensorimotor coverage, and use prospectively powered analyses with pre-specified confounders.

## 5. Conclusions

During eyes-closed stance on foam, fatigue in MS seems linked to stronger, right-biased activation of prefrontal and temporo-parietal cortices. Further studies on a larger sample may elucidate the presence of a distinctive pattern of connectivity which is globally reduced yet locally increased in BA10. These findings fit a compensatory-control account of balance under sensory challenge and motivate biomarker-driven trials to distinguish compensatory from restorative neural change in rehabilitation.

## Figures and Tables

**Table 1:** Clinical and demographic characteristics of fatigued and non-fatigued PwMS. EDSS: Expanded Disability Status Score, ABC: Activity-specific Balance Confidence Scale; T25FW: Timed 25-Foot Walk; PwMS: People with Multiple Sclerosis.

	Non-Fatigued PwMS			Fatigued PwMS		
	Mean ± SD	Min–Max	Range	Mean ± SD	Min–Max	Range
Age [year]	42.1 ± 11.0	27.0–58.0	31.0	48.7 ± 5.9	42.0–58.0	16.0
Education [year]	15.1 ± 3.7	10.0–23.0	13.0	13.0 ± 2.9	8.0–18.0	10.0
EDSS [score]	1.7 ± 0.9	0.0–3.0	3.0	2.7 ± 0.7	1.5–4.0	2.5
mini-BESTest	26.3 ± 1.7	23.0–28.0	5.0	21.3 ± 1.8	20.0–25.0	5.0
ABC [%]	87.2 ± 17.1	38.1–99.4	61.2	67.2 ± 11.7	53.1–87.5	34.4
T25FW [s]	4.1 ± 0.7	3.4–5.8	2.4	5.6 ± 0.8	4.3–6.7	2.4
Perimeter [mm]	427.8 ± 115.0	261.7–645.4	383.6	516.8 ± 119.7	366.2–743.9	377.7

## Data Availability

The datasets analyzed during the current study are available from the corresponding author on reasonable request.

## Supplementary Materials

The following supporting information can be downloaded at: https://www.mdpi.com/article/10.3390/s26165131/s1, Supplementary Material S1: Summary of MNI coordinates and Brodmann Areas of channel position, computed as midpoint of corresponding source and detector indexes.; Supplementary Material S2: Model 1 details; Supplementary Material S3: Model 2 details.

## Abbreviations

The following abbreviations are used in this manuscript:

ABC	Activity-specific Balance Confidence Scale
ANOVA	Analysis of variance
AR(1)	First-order autoregressive
BA	Brodmann area
CoP	Center of pressure
EC	Eyes closed
EDSS	Expanded Disability Status Scale
EEG	Electroencephalography
EO	Eyes open
fNIRS	Functional near-infrared spectroscopy
HbO	Oxy-hemoglobin
HbR	Deoxy-hemoglobin
HC	Healthy controls
HR	Hemodynamic response
IQR	Interquartile range
MFIS	Modified Fatigue Impact Scale
Mini-BESTest	Mini-Balance Evaluation Systems Test
MNI	Montreal Neurological Institute
PCA	Principal component analysis
PwMS	People with multiple sclerosis
RMSE	Root-mean-square error
SNR	Signal-to-noise ratio
T25FW	Timed 25-Foot Walk
