# Supplementary material for: Brain Activity and Connectivity in Fatigued People with Multiple Sclerosis During a Static Balance Task: An fNIRS Study
Source: Sensors (Basel). 2026 Aug 13;26(16):5131. doi: 10.3390/s26165131 (PMC13517440; doi:10.3390/s26165131)
Supplement: Supplementary file 1 [file sensors-26-05131-s001.zip › sensors-4431256-supplementary.pdf]

Supplementary Material S1: Summary of MNI coordinates and Brodmann Areas of channel position, computed as midpoint of corresponding source and detector indexes.

	MNI coords				
**channel**	**X_{MNI}**	**Y_{MNI}**	**Z_{MNI}**	**BA**	**Side**
**S1 - D1**	-33	46	1	BA10	Left
**S1 - D2**	-28	61	0	BA10	Left
**S2 - D1**	-37	54	9	BA10	Left
**S2 - D2**	-23	66	4	BA10	Left
**S2 - D3**	-10	63	19	BA10	Left
**S3 - D2**	-12	69	-1	BA10	Left
**S3 - D3**	3	61	12	BA10	Right
**S3 - D4**	15	70	-2	BA10	Right
**S4 - D3**	15	71	23	BA10	Right
**S4 - D4**	24	53	4	BA10	Right
**S4 - D5***	45	62	13	BA10	Right
**S5 - D4**	27	55	2	BA10	Right
**S5 - D5**	47	53	1	BA10	Right
**S6 - D6**	55	-14	10	BA40	Right
**S6 - D7**	51	-26	-6	BA21-22	Right
**S7 - D6**	47	-27	26	BA40	Right
**S7 - D7**	62	-44	12	BA21 22	Right
**S7 - D8**	56	-50	27	BA39	Right
**S8 - D7**	66	-51	-7	BA21-22	Right
**S8 - D8**	44	-59	16	BA39	Right
**S8 - D16**	52	-74	-1	BA19	Right
**S9 - D9**	-51	-17	8	BA40	Left

	MNI coords				
**channel**	**X_{MNI}**	**Y_{MNI}**	**Z_{MNI}**	**BA**	**Side**
**S9 - D10**	-51	-32	-8	BA21-22	Left
**S10 - D9**	-65	-28	32	BA40	Left
**S10 - D10**	-58	-47	8	BA21-22	Left
**S10 - D11**	-51	-54	29	BA39	Left
**S11 - D10**	-62	-56	-8	BA21-22	Left
**S11 - D11**	-35	-60	10	BA19	Left
**S11 - D13**	-42	-74	-3	BA19	Left
**S12 - D11**	-45	-72	34	BA39	Left
**S12 - D12**	-27	-73	35	BA40	Left
**S12 - D13**	-42	-85	18	BA19	Left
**S13 - D12***	-21	-95	21	BA18	Left
**S13 - D13**	-20	-89	-2	BA18	Left
**S13 - D14**	-9	-99	7	BA18	Left
**S14 - D12**	-15	-83	37	BA19	Left
**S14 - D14**	3	-95	25	BA18	Right
**S14 - D15**	22	-85	41	BA19	Right
**S15 - D14**	18	-98	8	BA18	Right
**S15 - D15**	26	-89	15	BA18	Right
**S15 - D16**	34	-94	1	BA18	Right
**S16 - D8**	49	-68	32	BA39	Right
**S16 - D15**	29	-68	32	BA40	Right
**S16 - D16**	43	-74	18	BA19	Right

Supplementary Material S2

1.1. Model specification

Item	Description
Outcome	HbO concentration (μM)
Fixed effects	BA * Group * Side
Random effects	~1 | ID
Random-effects SD (Intercept)	0.24
Residual SD (sigma)	0.094
Correlation structure	corARMA
Correlation formula	~time | SBJ/channel
Correlation parameters	0.986333
Estimation method	REML

HbO = oxygenated hemoglobin; BA = Brodmann area; Side = cortical hemisphere (Left/Right); SD = residual standard deviation; corARMA = autoregressive moving-average correlation structure; REML = restricted maximum likelihood; "~time | SBJ/channel" = correlation modeled across repeated time points within each subject and channel.

1.2. Model fit indices

AIC	BIC	R2 (conditional)	R2 (marginal)	ICC
-339,409.5	-339,076.4	0.91	0.31	0.869

AIC = Akaike information criterion (lower values indicate better relative fit for models fitted to the same data); BIC = Bayesian information criterion (stricter penalty for model complexity; lower is better); R^2 (conditional) = variance explained by fixed + random effects; R^2 (marginal) = variance explained by fixed effects only; ICC = intraclass correlation coefficient (proportion of variance attributable to between-subject differences).

1.3 Type III ANOVA tests

Effect	numDF	denDF	F_value	p_value
(Intercept)	1	178,974	10.218	0.0014
BA	4	178,974	315.729	<.0001
Group	2	32	3.532	0.0411
Side	1	178,974	2.903	0.0884
BA:Group	8	178,974	134.720	<.0001
BA:Side	4	178,974	34.775	<.0001
Group:Side	2	178,974	11.624	<.0001
BA:Group:Side	8	178,974	19.981	<.0001

Tests are Type III F-tests of fixed effects from the REML-fitted mixed. numDF = numerator degrees of freedom; denDF = denominator degrees of freedom.

Factors: Group = HS, f_PwMS, and nf_PwMS; BA = Brodmann area; Side = hemisphere (Left/Right).

1.4. Fixed effects (full)

Term	Estimate	SE	DF	t	p_value
(Intercept)	0.068221	0.056086	178,974	1.216	0.2238
BA18	0.103700	0.018072	178,974	5.738	<.0001
BA19	0.030356	0.014267	178,974	2.128	0.0334
BA21-22	0.069604	0.015677	178,974	4.440	<.0001
BA39-40	0.172422	0.015403	178,974	11.194	<.0001
f_PwMS	0.456675	0.114436	32	3.991	0.0004
nf_PwMS	0.052587	0.095472	32	0.551	0.5856
Right	0.054195	0.013097	178,974	4.138	<.0001
BA18:f_PwMS	-0.600928	0.036179	178,974	-16.610	<.0001
BA19:f_PwMS	-0.438263	0.029247	178,974	-14.985	<.0001
BA21-22:f_PwMS	-0.097927	0.032408	178,974	-3.022	0.0025
BA39-40:f_PwMS	-0.362973	0.032108	178,974	-11.305	<.0001
BA18:nf_PwMS	-0.598376	0.030468	178,974	-19.640	<.0001

Term	Estimate	SE	DF	t	p_value
BA19:nf_PwMS	-0.192571	0.024070	178,974	-8.001	<.0001
BA21-22:nf_PwMS	-0.106481	0.026444	178,974	-4.027	<.0001
BA39-40:nf_PwMS	-0.049568	0.026169	178,974	-1.894	0.0582
BA18:Right	-0.191952	0.023272	178,974	-8.248	<.0001
BA19:Right	-0.091046	0.021151	178,974	-4.305	<.0001
BA21-22:Right	-0.031724	0.022181	178,974	-1.430	0.1526
BA39-40:Right	-0.160391	0.020996	178,974	-7.639	<.0001
f_PwMS:Right	0.020056	0.025859	178,974	0.776	0.4380
nf_PwMS:Right	0.030089	0.021963	178,974	1.370	0.1707
BA18:f_PwMS:Right	-0.000749	0.046495	178,974	-0.016	0.9872
BA19:f_PwMS:Right	0.177156	0.043007	178,974	4.119	<.0001
BA21-22:f_PwMS:Right	-0.079477	0.045678	178,974	-1.740	0.0819
BA39-40:f_PwMS:Right	0.183648	0.043730	178,974	4.200	<.0001
BA18:nf_PwMS:Right	0.233588	0.039116	178,974	5.972	<.0001
BA19:nf_PwMS:Right	-0.059311	0.035952	178,974	-1.650	0.0990
BA21-22:nf_PwMS:Right	-0.079362	0.037408	178,974	-2.122	0.0339
BA39-40:nf_PwMS:Right	-0.140140	0.035628	178,974	-3.933	<.0001

SE = standard error; Factor coding: Group reference = HS; BA reference = BA10; Side reference = Left. Terms separated by colons (e.g., f_PwMS:BA39-40:Right) denote interaction effects.

Supplementary Material S3

2.1. Model specification

Item	Description
Outcome	HbO concentration (µM)
Fixed effects	Perimeter : BA * Group * Side
Random effects	~1 | SBJ
Random-effects SD (Intercept)	0.25
Residual SD (sigma)	0.094
Correlation structure	corARMA
Correlation formula	~time | SBJ/channel
Correlation parameters	0.986371
Estimation method	REML

HbO = oxygenated hemoglobin; BA = Brodmann area; Side = cortical hemisphere (Left/Right); SD = residual standard deviation; corARMA = autoregressive moving-average correlation structure; REML = restricted maximum likelihood; "~time | SBJ/channel" = correlation modeled across repeated time points within each subject and channel.

2.2 Model fit indices

AIC	BIC	R2 (conditional)	R2 (marginal)	ICC
-338,934.2	-338,540.5	0.915	0.315	0.876

AIC = Akaike information criterion (lower values indicate better relative fit for models fitted to the same data); BIC = Bayesian information criterion (stricter penalty for model complexity; lower is better); R^2 (conditional) = variance explained by fixed + random effects; R^2 (marginal) = variance explained by fixed effects only; ICC = intraclass correlation coefficient (proportion of variance attributable to between-subject differences).

2.3 Type III ANOVA tests

Effect	numDF	denDF	F_value	p_value
(Intercept)	1	178,968	9.575	0.0020
Group	2	32	3.291	0.0502
Side	1	178,968	13.732	0.0002
Perimeter:BA	5	178,968	336.742	<.0001

Effect	numDF	denDF	F_value	p_value
Group:Side	2	178,968	6.882	0.0010
Perimeter:BA:Group	10	178,968	52.715	<.0001
Perimeter:BA:Side	5	178,968	34.621	<.0001
Perimeter:BA:Group:Side	10	178,968	24.710	<.0001

Tests are Type III F-tests of fixed effects from the REML-fitted mixed. numDF = numerator degrees of freedom; denDF = denominator degrees of freedom.

Factors: Group = HC, f_PwMS, and nf_PwMS; BA = Brodmann area; Side = hemisphere (Left/Right), Perimeter.

2.4. Fixed effects (full)

Term	Estimate	SE	DF	t	p_value
(Intercept)	0.191163	0.184200	178,968	1.038	0.2994
f_PwMS	-0.114323	0.270637	32	-0.422	0.6755
nf_PwMS	-0.100636	0.289660	32	-0.347	0.7305
Right	-0.033593	0.022732	178,968	-1.478	0.1395
Perimeter:BA10	-0.000079	0.000166	178,968	-0.475	0.6347
Perimeter:BA18	-0.000071	0.000166	178,968	-0.426	0.6698
Perimeter:BA19	-0.000106	0.000166	178,968	-0.639	0.5227
Perimeter:BA21-22	-0.000050	0.000166	178,968	-0.302	0.7627
Perimeter:BA39-40	0.000035	0.000166	178,968	0.210	0.8337
f_PwMS:Right	0.277673	0.033873	178,968	8.198	<.0001
nf_PwMS:Right	0.180940	0.035763	178,968	5.059	<.0001
Perimeter:BA10:f_PwMS	0.000348	0.000192	178,968	1.811	0.0701
Perimeter:BA18:f_PwMS	0.000086	0.000193	178,968	0.448	0.6544
Perimeter:BA19:f_PwMS	0.000137	0.000192	178,968	0.711	0.4768
Perimeter:BA21-22:f_PwMS	0.000212	0.000192	178,968	1.101	0.2708
Perimeter:BA39-40:f_PwMS	0.000103	0.000192	178,968	0.535	0.5925
Perimeter:BA10:nf_PwMS	0.000076	0.000238	178,968	0.320	0.7491

Term	Estimate	SE	DF	t	p_value
Perimeter:BA18:nf_PwMS	-0.000226	0.000239	178,968	-0.945	0.3448
Perimeter:BA19:nf_PwMS	0.000016	0.000239	178,968	0.068	0.9461
Perimeter:BA21-22:nf_PwMS	0.000049	0.000239	178,968	0.207	0.8359
Perimeter:BA39-40:nf_PwMS	0.000058	0.000239	178,968	0.245	0.8067
Perimeter:BA10:Right	0.000049	0.000023	178,968	2.168	0.0302
Perimeter:BA18:Right	-0.000051	0.000026	178,968	-1.963	0.0497
Perimeter:BA19:Right	0.000009	0.000024	178,968	0.379	0.7045
Perimeter:BA21-22:Right	0.000048	0.000025	178,968	1.905	0.0568
Perimeter:BA39-40:Right	-0.000048	0.000024	178,968	-1.977	0.0480
Perimeter:BA10:f_PwMS:Right	-0.000164	0.000027	178,968	-6.053	<.0001
Perimeter:BA18:f_PwMS:Right	-0.000241	0.000033	178,968	-7.241	<.0001
Perimeter:BA19:f_PwMS:Right	-0.000051	0.000031	178,968	-1.655	0.0980
Perimeter:BA21-22:f_PwMS:Right	-0.000148	0.000033	178,968	-4.467	<.0001
Perimeter:BA39-40:f_PwMS:Right	-0.000023	0.000031	178,968	-0.752	0.4520
Perimeter:BA10:nf_PwMS:Right	-0.000110	0.000033	178,968	-3.336	0.0009
Perimeter:BA18:nf_PwMS:Right	0.000010	0.000038	178,968	0.266	0.7901
Perimeter:BA19:nf_PwMS:Right	-0.000174	0.000036	178,968	-4.852	<.0001
Perimeter:BA21-22:nf_PwMS:Right	-0.000209	0.000037	178,968	-5.663	<.0001
Perimeter:BA39-40:nf_PwMS:Right	-0.000222	0.000036	178,968	-6.194	<.0001

SE = standard error. Terms separated by colons (e.g., f_PwMS:BA39-40:Right) denote interaction effects.
